# Changes in Use of a Leisure Activity Mobile App for Children With Disabilities During the COVID-19 Pandemic: Retrospective Study

**DOI:** 10.2196/32274

**Published:** 2022-02-25

**Authors:** Paul Yejong Yoo, Mehrnoosh Movahed, Ishana Rue, Carlos Denner Dos Santos, Annette Majnemer, Keiko Shikako

**Affiliations:** 1 Faculty of Medicine and Health Sciences McGill University Montreal, QC Canada; 2 Management Department University of Brasilia Brasilia Brazil; 3 Computer Science Department Ecole de Technologie Supérieure Montreal, QC Canada

**Keywords:** COVID-19, participation, childhood disability, online leisure, app engagement, mHealth, children, parents, mobile apps, mobile health, digital health, pandemic, online leisure activities, user engagement, app usability

## Abstract

**Background:**

Participation in leisure activities is essential for child development and a human right as per the United Nations Convention on the Rights of the Child. Children with disabilities face several restrictions when participating in leisure activities as compared to same age peers without disabilities. Access to information about accessible, inclusive leisure activities is one of the barriers limiting participation, and one potential health promotion strategy is to provide access to information to increase participation. The Jooay App is a mobile app listing such activities in Canada and Australia. With the COVID-19 global pandemic and subsequent public health measures, most community-based facilities providing the activities listed on Jooay were closed. The app therefore started listing online activities offered with the expectation of continuing to provide information for families and understanding the extent to which users relied on the mobile app as a tool to identify new safe leisure opportunities.

**Objective:**

This study aims to describe the engagement of the Jooay app before and during COVID-19, and to estimate the extent to which the listing of online activities was related to the engagement of the Jooay app.

**Methods:**

We conducted a retrospective study comparing Jooay app use between March 2020 and February 2021 to the engagement between March 2019 and February 2020 by Jooay users. Spearman rank correlations were carried out to identify associations between the activities listed and the users’ engagement from May 2020 to February 2021.

**Results:**

Active engagement with the Jooay app from March 2020 to February 2021 dropped by an average of 135 engagements (64.2%) compared to engagements in 2019-2020. The largest monthly drop in engagement was observed in May 2020 by 239 engagements (88.8%). There was a strong positive correlation between the number of active users and the number of online activities listed on the app (r_s_=0.900).

**Conclusions:**

The engagement with the Jooay App presented an expected decrease during the first wave of the COVID-19 pandemic. The addition of online adapted leisure activities to the app’s listings during the pandemic increased app use. Access to information about inclusive activities is a barrier for children with disabilities to engage in leisure. Mobile health solutions can be responsive to contextual factors and consider the social determinants of health such as socioeconomic and public health emergency issues that can impact the participation of vulnerable populations such as children with disabilities and help eliminate barriers to participation. The provision of online leisure opportunities during the pandemic could facilitate participation in these activities during the pandemic and beyond, which is essential and beneficial for the physical and mental well-being of children with disabilities and their families.

## Introduction

Participation of children with disabilities in leisure activities is a key determinant of their physical and mental health, and that of their families [1]. Participation in a variety of leisure activities is associated with quality of life [2]. Participation in leisure and play is also a human right as stated in the United Nations’ Convention on the Rights of the Child [3], and participation in the community is a key human right as articulated in the Convention on the Rights of Persons with Disabilities [4].

Despite the benefits and importance of participation, children with disabilities face participation restrictions in comparison to their peers who do not have a disability [5,6]. Several barriers contribute to this reduced participation, one of which is limited access to information about existing activities that meet the child and families’ needs [7,8]. In fact, families of children with disabilities and health care professionals indicated that information about existing resources and leisure opportunities that will include children with developmental challenges is difficult to find, requiring additional effort on the part of already overwhelmed parents. Additionally, existing activities often do not accommodate for the child’s disability needs or are not aligned with the child’s context (eg, are far from the house or the school) [9,10]. The Jooay App was launched in Spring 2015 to overcome this gap between families, health care providers, and community-based leisure activities. This mobile health (mHealth) solution lists inclusive and adapted leisure activities across Canada and Australia, providing free crowdsourced information about the characteristics of leisure activities offered based on geographical location.

With the COVID-19 global pandemic declared by the World Health Organization (WHO) in March 2020 [11], countries worldwide, including Canada, had to implement massive emergency public health stringency measures to protect the public with prevention protocols to limit the spread of the virus. These public health measures included self-isolation, social distancing, and stay-at-home recommendations in Canada [12]. With these measures in place, many public and private facilities were obliged to close, including most leisure activities listed on Jooay. Access to any form of leisure was made much more challenging for children with disabilities and their families, limiting essential opportunities for development and health promotion.

Lockdown restrictions following the COVID-19 pandemic had significant negative effects on the physical activity levels and mental and behavioral health of children with disabilities [13]. It was reported that these negative effects were brought on by the lack of access to facilities and activities [13]. With technology becoming essential during the COVID-19 pandemic, children need virtual opportunities to learn, live, and stay connected to maintain their physical, social, and mental well-being [14].

Before the COVID-19 pandemic, all leisure activities listed in Jooay were in-person activities requiring physical attendance. Online leisure activities began being offered as an alternative to provide some form of connection during the pandemic. In response, the Jooay App crowdsourced online activities being offered worldwide for children with disabilities on the Jooay app. As of February 2021, there were a total of 382 online activities listed.

The objective of this study was to explore the extent to which families of children with disabilities use a mobile app to access information about leisure activities for their child by understanding the changes in the pattern of use of a geolocation-based mHealth solution during the COVID-19 pandemic and to estimate the extent to which the listing of online activities was related to the users’ engagement with the Jooay app. It was hypothesized that parents do use mobile apps to find activities that are appropriate for their child, and as such, the availability of online activities on the app would be positively correlated with app engagement.

## Methods

### Study Design

This study was a retrospective study that compared the Jooay app engagement between March 2020 and February 2021, for a total of 12 months, to the engagement between March 2019 and February 2020 (prepandemic). March 2020 was chosen as the beginning of the timeframe of this study because it was the month when the WHO declared COVID-19 as a global pandemic and mass closures of public facilities started happening in Canada [11,15]. The STROBE (Strengthening the Reporting of Observational Studies in Epidemiology Statement) checklist was used to guide the reporting of this study [16]. This study was approved by the Research Ethics Board of McGill University as part of a larger study.

### Population and Sample

The population of this study was users of the Jooay mobile app in Canada. Users included youth with disabilities, parents of children with disabilities, educators, and health care providers. Users of the app provide consent to have their deidentified data analytics used for research purposes upon registration with the app. Data on Jooay app engagement was collected each month within the time frame previously listed.

### Jooay App

Jooay is a free mobile and web-based app that helps children with disabilities and their families locate leisure opportunities that are inclusive and accessible, are in the communities where they live, suit their needs and abilities, match their preferences, and can help them develop and participate in society [17]. Jooay is also a social platform to help parents, rehabilitation professionals, educators, and communities connect, exchange, and learn from each other’s experiences. Currently, as of February 28, 2021, Jooay lists 3250 activities across all 10 Canadian provinces and 1 territory (Yukon), with 3124 total users. As of March 2021, Jooay is also available in Australia. This study reports on app use in Canada only.

### Measures: Variables

Two variables were investigated for this study. The first variable was the number of online leisure activities available on the Jooay app; this is a discrete variable (ie, count). The addition of online leisure activities started in May 2020, 2 months after the declaration of a pandemic in March 2020 and continued to be updated and expanded until February 2021. The second variable was app engagement over the determined periods of time. App engagement was determined by the number of unique users that activated a session on the Jooay app in the specified month [18].

### Data Analysis

Descriptive statistics were performed using Excel software (Microsoft Corporation). App engagement was compared in the 12-month period of the COVID-19 pandemic (March 2020 to February 2021) to the engagement in the corresponding months before the pandemic (March 2019 to February 2020). Spearman rank correlation analysis was conducted using SPSS 27 (IBM Corp) between the number of online activities available to users and the app engagement from May 2020 to February 2021, as previously defined, to estimate the relationship between the two variables.

## Results

### User Characteristics

As of February 28, 2020, there were 3124 registered users of the Jooay app. Of those who have reported their sex (n=936), 627 (67%) were female and 309 (33%) were male. Participants’ characteristics are described in Table 1. Users were asked to select the category that best represented who they were (eg, parent, health professional, or educator) and the classification of disability that best described the individuals they were seeking activities for (could select more than one). The majority of participants were parents of children with disabilities, and their children had a range of disabilities, predominantly autism spectrum disorder or physical disabilities, though 37.7% (n=1400) of participants did not select one specific disability type.

**Table 1:** User characteristics.

Characteristics		Users, n (%)
**User types (n=3124)**		
	Parents	1260 (40.3)
	Health care professionals	656 (21.0)
	Other	267 (8.5)
	Community organizations	251 (8.0)
	Educators	222 (7.1)
	Individuals	167 (5.3)
	Not disclosed^a^	301 (9.6)
**Disability^b^ (n=3714)**		
	Autism spectrum disorder	660 (17.8)
	Physical	651 (17.5)
	Intellectual	524 (14.1)
	Behavioral	234 (6.3)
	Visual	141 (3.8)
	Auditory	104 (2.8)
	Not disclosed^a^	1400 (37.7)

^a^Not disclosed users are users who did not report their user type.

^b^A single user could report multiple disability types.

### Activity Characteristics

The Jooay app has activities across 10 different provinces, with a higher concentration of activities listed in urban centers, and the majority of activities listed in the largest provinces (Ontario and Quebec). The types of activities are categorized as arts, sports, camps, and other on the app; the other category includes, for example, activities like life skills activities, money management, respite, and cooking. The distribution of activities listed per province can be found in Table 2.

**Table 2:** Activity characteristics (N=3250)^a^.

Province	Activity type, n (%)			
	Arts	Camps	Sports	Other
Ontario (n=627)	71 (11.3)	187 (29.8)	209 (33.3)	160 (25.5)
Quebec (n=530)	56 (10.5)	122 (23.0)	181 (34.2)	171 (32.3)
Alberta (n=333)	40 (12.0)	45 (13.5)	161 (48.3)	87 (26.1)
British Columbia (n=227)	9 (4.0)	25 (11.0)	130 (57.3)	63 (27.8)
Manitoba (n=124)	21 (16.9)	17 (13.7)	67 (54.0)	19 (15.3)
Saskatchewan (n=124)	8 (6.5)	33 (26.6)	31 (25.0)	52 (41.9)
Prince Edward Island (n=109)	9 (8.3)	20 (18.3)	52 (47.7)	28 (25.7)
New Brunswick (n=94)	6 (6.4)	29 (30.9)	31 (33.0)	28 (29.8)
Nova Scotia (n=84)	11 (13.1)	22 (26.2)	29 (34.5)	22 (26.2)
Newfoundland and Labrador (n=44)	5 (11.4)	1 (2.3)	32 (72.7)	6 (13.6)
Yukon (n=15)	0 (0.0)	0 (0.0)	10 (66.7)	5 (33.3)

^a^Of these 3250 activites, 939 were activities in Australia, activities across Canada, and activities without specified provinces.

### Jooay App Engagement

**Figure 1:**
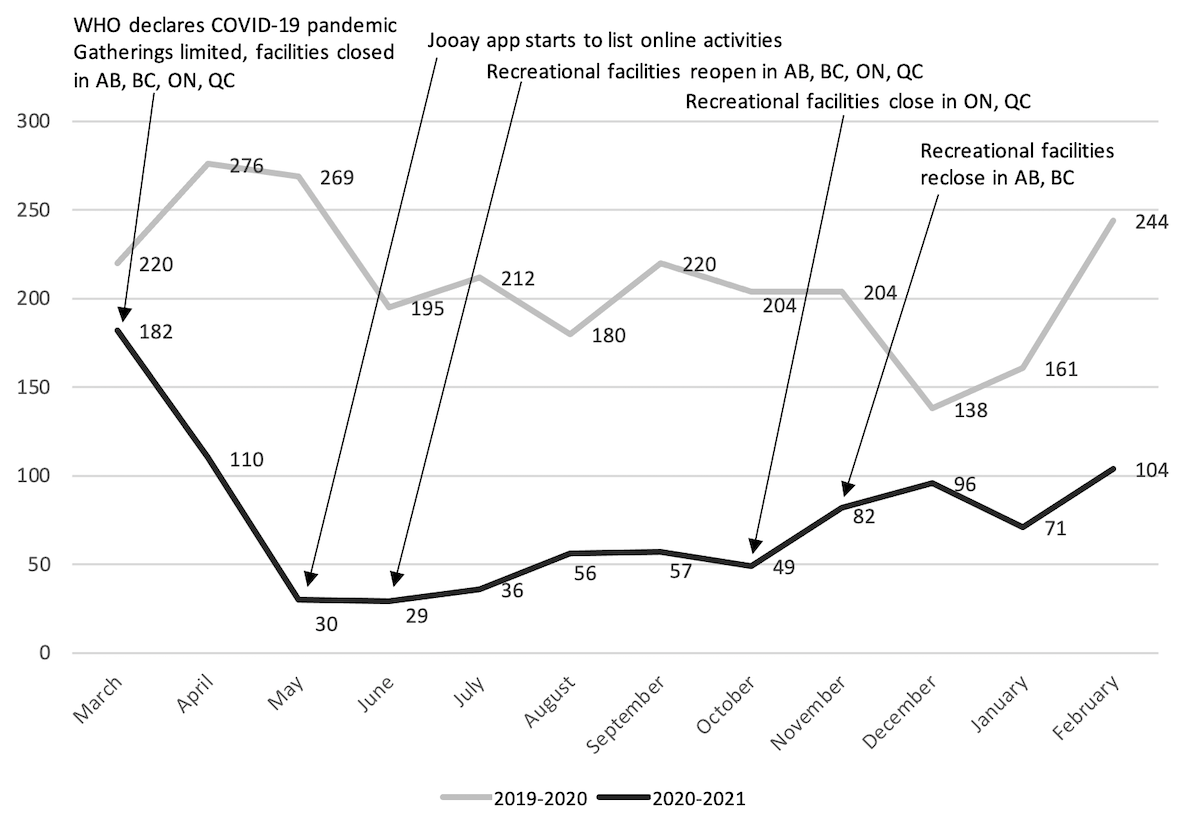
The number of monthly active app engagements across the two different time periods and corresponding COVID-19 pandemic directives. WHO: World Health Organization.

Figure 1 illustrates the app engagement pattern for the periods of March 2019 to February 2020 (prepandemic) and that of March 2020 to February 2021 (pandemic). There is a predictable trend in app engagement for the 2019-2020 period. Higher number of app engagements were observed in April and May 2019, being the months preceding registration for summer activities; then again in September, the time for registration for Fall activities; and then finally in February, the time for registration for activities during March break in most Canadian grade schools.

Figure 1 also presents the monthly total app engagements during the main events of the COVID-19 pandemic between March 2020 and February 2021. The active engagement of the Jooay app from the months of March 2020 to February 2021 dropped an average of 135 engagements per month (64.2%) compared to engagements by users during that same period in 2019-2020. The greatest monthly drop in engagement was observed in May 2020, with a drop of 239 monthly active users compared to that of 2019, which was a drop of 88.8%. The lowest drop in engagement was observed in March 2020, with a drop of 38 monthly active users compared to that of 2019, a 17.3% decrease.

### Availability of Online Activities

**Figure 2:**
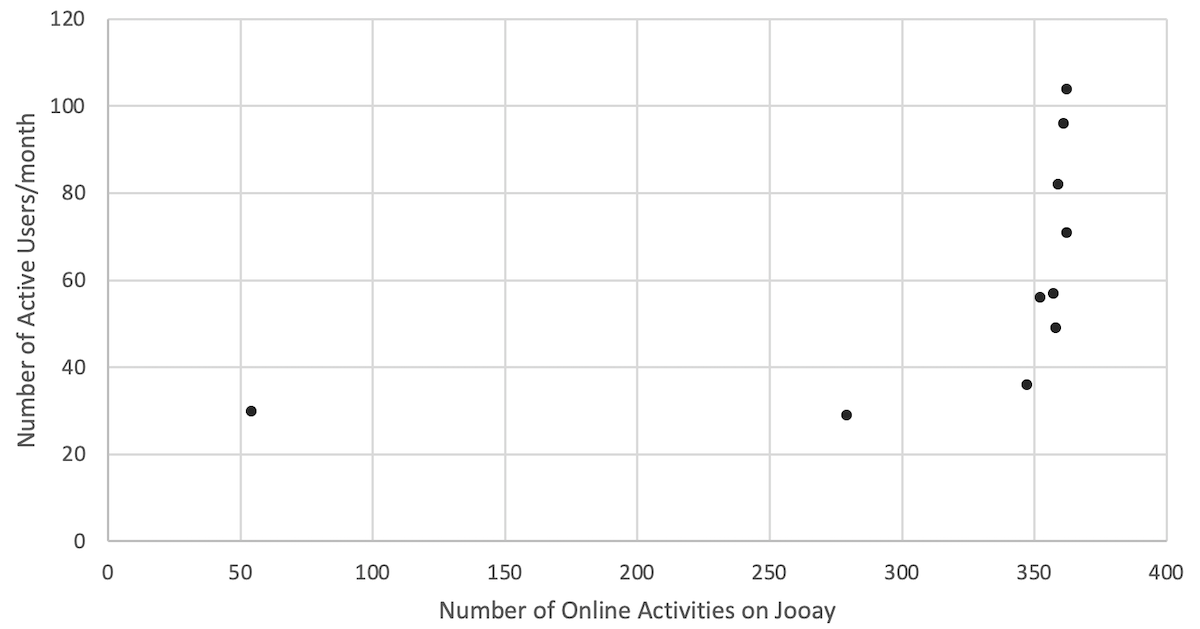
Online activities on the Jooay app between May 2020 and February 2021.

**Figure 3:**
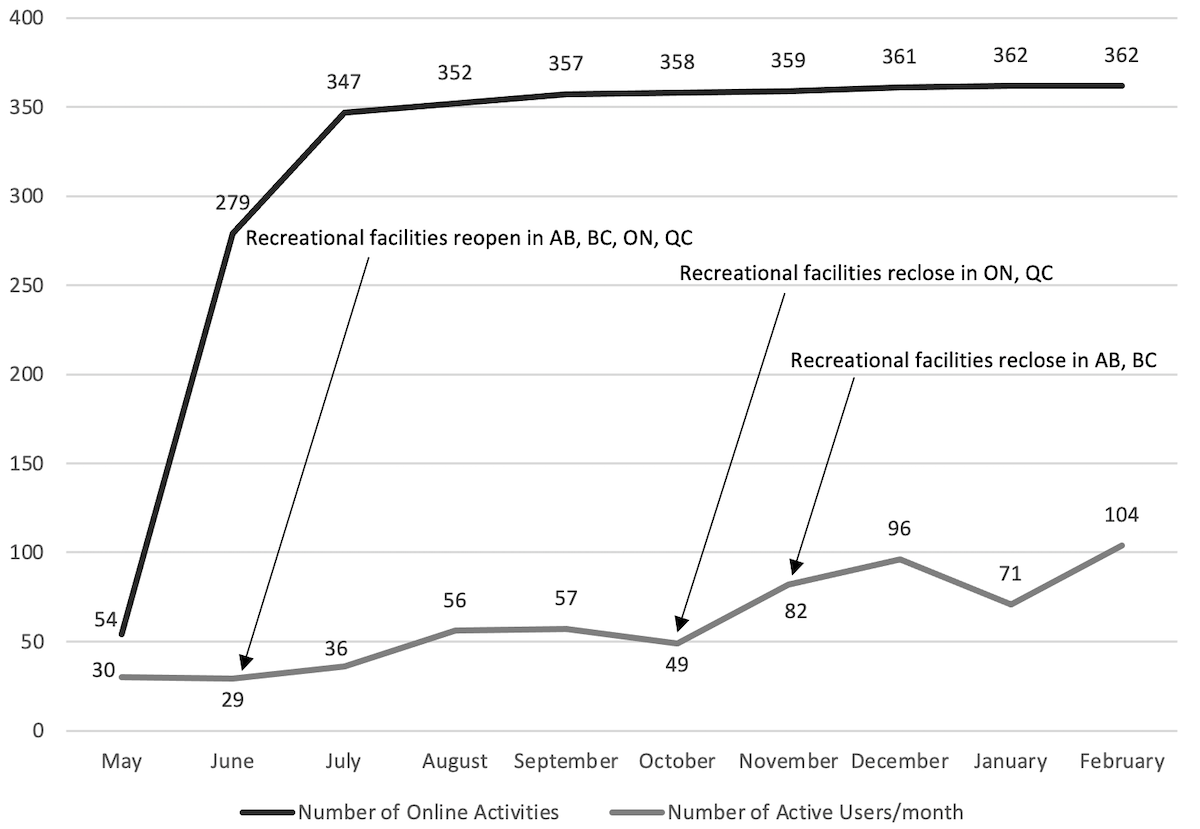
The number of online activities on the Jooay app and the number of monthly active users from May 2020 to February 2021.

Online activities were posted on the Jooay app starting in May 2020 up until February 2021. Following the addition of online activities on the app, the number of active users gradually increased (Figure 2). There was a strong positive correlation between the number of monthly app engagements and the number of online activities listed on the app at that time (r_s_=0.900). Figure 3 presents the number of online activities available on the app and the active users.

## Discussion

### Principal Results

This study presents the use patterns of an mHealth app targeting adapted and inclusive leisure activities for children with disabilities and describes changes that were observed during the COVID-19 pandemic and the subsequent health measures that were implemented in Canada [11]. This study shows an average drop in the monthly active engagement of the app by 135 engagements (64.2%) from the months of March 2020 to February 2021 compared to that of the same period in 2019-2020. This drop in engagement likely relates to closures and ongoing insecurity in the offering of community activities, possibly reflecting a gap in the existing opportunities for physical and leisure activities for children worldwide during this pandemic.

The global spread of COVID-19 led to unprecedented governmental measures that resulted in many restrictions in Canada. As a result, organizations providing services and activities for children with disabilities were forced to close, along with other sectors of society. Data from Statistics Canada and a WHO report on disabilities demonstrate that indeed parents of children with disabilities identified the lack of leisure opportunities, along with school closures and limited health care services as critical components for their child’s health and well-being during the pandemic [19].

On March 11, 2020, the WHO declared the COVID-19 outbreak to be a global pandemic [11]. A day before this declaration, Canada published the recommendation for the adoption of work-from-home policies and the guidance on self-isolation [20]. This acted as a reinforcement for the provincial policies that were being rolled out and as groundwork for future provincial health policies. In Canada, although the health care system is largely determined by the Canadian Constitution, many of the roles and mandates of delivering health and social services fall under provincial jurisdiction [21]. This includes the planning and implementation of public health initiatives [21].

In the time frame of this study, the majority of activities listed on the Jooay app were found within the four largest provinces in Canada: Ontario, Quebec, British Columbia (BC), and Alberta. In all four of these provinces, recreational facilities and entertainment venues were required to close, while gatherings of more than five people in Ontario, more than 15 people in Alberta, and more than 50 people in BC were prohibited; in Quebec, gatherings of any size were prohibited, while BC recommended avoidance of gatherings of any size in March [20]. The smallest decrease in the monthly app engagement was observed in March by 38 engagements (17.3%). This may be due to engagement that was not impacted in the first half of the month and the immediacy of the implementation of the measures.

The greatest decrease in the monthly app engagement was observed in the month of May, with engagement for that month dropping by 239 engagements (88.8%) in 2020 compared to 2019. With the end of the academic year and the start of the summer season approaching, May has traditionally been the month of high app use, during which parents and families of children with disabilities use the Jooay app to search for activities/camps that are scheduled for the summer of that year. However, with the residual fears and uncertainty of COVID-19 and the restrictions in place in May 2020, the decrease in app engagement can be understood. Closures and restrictions were maintained until June 2020; the number of people allowed in gatherings gradually increased and facilities reopened in many provinces around this time. Even with the eased measures, the engagement of the app did not change. These use patterns can shed light into important features of mobile app use as a source of information for “secondary health outcomes.” In times of crises like this, children with disabilities are likely to face greater challenges due to attitudinal, environmental, and institutional barriers [22,23]. These barriers often stem from the lack of governmental response and the persistent neglect of this vulnerable group by society [22]. In the immediate responses to the pandemic, public health measures and governmental support were not necessarily appropriately targeted to the needs of the disability community and much less to the needs of children with disabilities [24]. No specific resources nor support were provided for children with disabilities in the first year of the pandemic by the federal government, while parents and families of children with disabilities reported high concern for the well-being of their child during the pandemic [19]. Families of children with disabilities and the children themselves reported heightened levels of stress and anxiety surrounding the COVID-19 pandemic; coupled with the increased responsibility for parents to be home with the children, these families felt overwhelmed [25]. This experience of high stress and uncertainty, as well as the overwhelming demands of providing immediate care for their child at home, may explain the continued low engagement of the Jooay app in summer 2020, despite the eased restrictions. The access to online activities listed in the app as of summer 2020 may have helped families identify new safe venues for their children, but at a point of distress, a geolocation-based app may not be consulted. Previous studies found that fear linked with insufficient knowledge about the pandemic has led to a lack of understanding and incorrect decision-making by parents [26]. It could be understood that the parents just did not have the resources, support, and sufficient knowledge about the ever-evolving situation to use the app. The use of a mobile app to provide information for vulnerable groups is not largely explored and represents an untapped potential for future research.

In response to the COVID-19 pandemic and the closures of facilities following health measures, online leisure activities were listed in the Jooay app starting in May 2020. Although the overall engagement of the app decreased by an average of 64.2% in the year 2020 compared to 2019, this study shows that as more online leisure activities were made available in the Jooay app, the number of active monthly users also increased. The number of users gradually increased, possibly because users were not aware that online activities were being added to the app, and this took time to gradually disseminate. According to Statistics Canada, more than 60% of parents reported concerns with their general mental health, loneliness, and lack of opportunities to socialize for their children [19]. In addition, 50% of parents were concerned about their children’s amount of physical activity. Therefore, the positive association between the number of online activities listed and greater Jooay app engagement can be explained in part by parent’s taking action to address these pressing concerns. A previous study found that activities listed in the app are less present in areas with lower socio and material deprivation, meaning that the number of offerings of activities is already skewed to urban centers and areas with higher population density. Closures of facilities and subsequent cancellation of in-person activities undoubtedly decreased opportunities for children to participate. Although online activities listed in the app offer an alternative to in-person activities, the lack of access to a mobile phone and to a computer and the internet poses an extra layer of inequity in the access to services, which was also exacerbated during the pandemic. Virtual platforms have become essential during the COVID-19 pandemic; it has become a solution through which children can play and interact with other peers [14]. Although the Statistics Canada report showed that approximately 70% of parents were concerned with the amount of screen time experienced by their child, technology, when accessible and available, allowed children to maintain their social, physical, emotional, intellectual, and spiritual well-being during the COVID-19 pandemic [19].

### Practical Implications

The continual offering of online leisure opportunities by service providers and organizations could help facilitate leisure participation of children with disabilities. The Jooay app will indeed continue to list online leisure opportunities. Furthermore, future health measures and policies should proactively consider the potential effects of those measures on marginalized groups such as children of the disability community by involving youth with disabilities and their families in the development and implementation of those measures and include equity and access considerations in the development of both emergency and continuous provision of services.

The combined use of mHealth solutions such as the Jooay app and other online platforms that present opportunities of essential activities such as education and leisure, provide an interesting future model to consider in the promotion of health, social inclusion, and equity. The provision of online resources for children was made an essential matter during the pandemic, accelerating the mitigation of digital access issues that persisted for many decades [27]. This study presents the reactions and habits of stakeholders in the childhood disability community, including families of children with disabilities, following a major health emergency and subsequent measures. It offers insight into how health measures and policy can affect their engagement with an online platform and how that engagement can change with the provision of online leisure opportunities. The study highlights the necessity of including marginalized groups, such as the disability community, and children and youth in the development of new solutions during emergency responses and beyond [28]. We can also understand that families do use mobile apps to find relevant information for services for their children, and therefore, this is a resource that should be further explored to decrease barriers to participation in health promotion initiatives.

### Limitations

This study presents the active engagement of Jooay, a free mobile and web-based app that helps children with disabilities and their families locate leisure opportunities that are inclusive and accessible in the communities where they live [17]. This noncommercial app has had exclusively word of mouth dissemination, and the data in the app is crowdsourced and may not be representative of leisure participation patterns both prior to and during the pandemic. The engagement does not directly translate into participation of the child with disability and does not determine if the child did indeed participate in a specific activity. However, access to information about leisure activities has been found to be a facilitator to participation [1]. The study also does not take into consideration the increase in the overall number of users over time, which may have an influence on the number of active users.

### Comparison With Prior Work

No prior work that is similar to this study has been conducted in the literature. However, the literature does show that the COVID-19 pandemic has caused an abrupt change worldwide while significantly affecting the lives of children with disabilities [29]. A recent sentiment analysis conducted by Boon-Itt and Skunkan [30] using Twitter data showed an overall negative emotion toward COVID-19, with *fear* as the most negative sentiment expressed [30]. Despite the scarce literature on the use of online/virtual environments for leisure activities for children with disabilities, the parent perspectives of pediatric telehealth during the pandemic, where therapeutic activities were provided on an online platform or by telephone, were discussed in a recent study [31]. Parents found that the greater need for management of technology and exacerbated feelings of isolation were disadvantages. However, many more advantages were reported, including emphasis on building relationships and rapport, less barriers when doing activities in the natural (home) environment, less travel, more time in the day to schedule other things, continuity of care, and decreased stress about physical interaction during the pandemic [31]. The perceived advantages of telehealth may have also been realized with the increased engagement of the Jooay app once online activities were listed for consideration. Furthermore, a recent study by Rovetta and Bhagavathula [32] presenting the global infodemiology of COVID-19 during the pandemic showed that there is a growing number of people worldwide who are using online search engines like Google to acquire information surrounding COVID-19 and to also disseminate information related to COVID-19 using social media platforms like Instagram [32]. Online activity, engagement, and presence is something that is being observed, as the COVID-19 pandemic and its restrictions have limited the availability of other methods of communication and information transfer. Future research should study the effects of the COVID-19 pandemic on the actual participation of children with disabilities in leisure activities. This should include a focus on the benefits and challenges of online leisure activities on the participation of children with disabilities.

### Conclusion

The global COVID-19 pandemic has led to restrictions and closures in local communities, including leisure activities that are important for child health. User engagement of Jooay, a free mobile and web-based app listing adapted and inclusive leisure opportunities for children with disabilities, decreased during the pandemic. However, the subsequent listing of online activities on the app had a strong positive correlation with the gradual increase in engagement of the Jooay app once a large number of online activities appropriate for children with disabilities were added. The Jooay app has the potential to be a medium through which children with disabilities and their families can readily access information and resources related to online leisure activities in which children can participate. The provision of online leisure opportunities during the pandemic could lead to increased participation, which is essential and beneficial for the physical and mental well-being of children with disabilities and their families.

## Abbreviations

BC: British Columbia
mHealth: mobile health
STROBE: Strengthening the Reporting of Observational Studies in Epidemiology Statement
WHO: World Health Organization
